# Genome-Wide Association Analysis Coupled With Transcriptome Analysis Reveals Candidate Genes Related to Salt Stress in Alfalfa (*Medicago sativa* L.)

**DOI:** 10.3389/fpls.2021.826584

**Published:** 2022-02-03

**Authors:** Fei He, Chunxue Wei, Yunxiu Zhang, Ruicai Long, Mingna Li, Zhen Wang, Qingchuan Yang, Junmei Kang, Lin Chen

**Affiliations:** Institute of Animal Science, Chinese Academy of Agricultural Sciences, Beijing, China

**Keywords:** alfalfa, GWAS, salt stress, RNA-seq, auxin

## Abstract

Salt stress is the main abiotic factor affecting alfalfa yield and quality. However, knowledge of the genetic basis of the salt stress response in alfalfa is still limited. Here, a genome-wide association study (GWAS) involving 875,023 single-nucleotide polymorphisms (SNPs) was conducted on 220 alfalfa varieties under both normal and salt-stress conditions. Phenotypic analysis showed that breeding status and geographical origin play important roles in the alfalfa salt stress response. For germination ability under salt stress, a total of 15 significant SNPs explaining 9%–14% of the phenotypic variation were identified. For tolerance to salt stress in the seedling stage, a total of 18 significant SNPs explaining 12%–23% of the phenotypic variation were identified. Transcriptome analysis revealed 2,097 and 812 differentially expressed genes (DEGs) that were upregulated and 2,445 and 928 DEGs that were downregulated in the leaves and roots, respectively, under salt stress. Among these DEGs, many encoding transcription factors (TFs) were found, including *MYB*-, *CBF*-, *NAC-*, and *bZIP-*encoding genes. Combining the results of our GWAS analysis and transcriptome analysis, we identified a total of eight candidate genes (five candidate genes for tolerance to salt stress and three candidate genes for germination ability under salt stress). Two SNPs located within the upstream region of *MsAUX28*, which encodes an auxin response protein, were significantly associated with tolerance to salt stress. The two significant SNPs within the upstream region of *MsAUX28* existed as three different haplotypes in this panel. Hap 1 (G/G, A/A) was under selection in the alfalfa domestication and improvement process.

## Introduction

Soil salinization is one of the major abiotic stresses limiting agricultural production in many areas of the world (Machado and Serralheiro, 2017). As a global environmental problem, soil salinization is mainly caused by irrigation and natural mineralization (Jin et al., 2019). More than 800 million hectares of land worldwide are affected by soil salinization, which accounts for more than 6% of global land area (Shahid et al., 2018). According to the latest prediction, more than 50% of land will be affected by soil salinization by 2050 (Wang et al., 2003). Various types of ions, such as sodium, potassium, calcium, magnesium and chloride ions, can cause soil salinization (Haq et al., 2018). With respect to these ions, sodium chloride is the most abundant salt in the soil. Soil salinization can severely affect the growth and development of plants; for example, seeds cannot germinate, plant height decreases, the root system becomes shorter, and normal flowering and fruiting cannot be achieved, which eventually leads to a decline in yield and quality (Deinlein et al., 2014). Moreover, some salt-sensitive plants or crops can die even under slight soil salt stress. Modern molecular genetics has proven that the main reason for this phenomenon is that under salt stress, the normal physiological and metabolic functions of plants are damaged by ionic, osmotic and oxidative stress (Zhang et al., 2010).

Alfalfa (*Medicago sativa* L.), an important legume crop species, plays an important role in improving saline-alkali soil and agricultural production because this species can fix atmospheric nitrogen (Shen et al., 2020). However, severe salt stress greatly reduces the growth and productivity of alfalfa. Therefore, the development of salt-tolerant alfalfa varieties is urgently needed for the sustainable production of alfalfa worldwide.

In the long-term evolutionary process, plants, especially some salt-tolerant plants, have developed adaptive mechanisms to address damage caused by salt stress (Zhao et al., 2020). Previous studies on model plant species such as Arabidopsis, rice and maize have revealed that transcription factors (TFs) and microRNAs play a large role in salt tolerance (Kreps et al., 2002 and Zhao et al., 2020). These molecular modules form a large gene regulatory network in plants to cope with salt stress (Zelm et al., 2020). Within this gene regulatory network, the plant response to high salt stress involves a variety of signal transduction pathways, including two main ones: an abscisic acid (ABA)-dependent pathway and an ABA-independent pathway. In the ABA-dependent pathway, many TFs, such as *MYB* (Dubos et al., 2010; Li J. et al., 2019 and Zhao et al., 2019), *bZIP* (Zhang M. et al., 2020; Bi et al., 2021), *NAC* (Joshi et al., 2016; Khedia et al., 2018; Li et al., 2021; Zhang X. et al., 2021) TFs, which have ABA-responsive element-binding factors in their promoter regions, play an important role in the plant response to salt stress. For example, Arabidopsis NAC069 mutants are insensitive to salt and cold stresses (He et al., 2017). Dehydration responsive element binding protein (DREB) TFs are involved in the salt stress response of plants mainly through the ABA-independent pathway (Zhao et al., 2016). Many studies have revealed that overexpression of C-repeat binding factor (CBF) enhances plant tolerance to salt stress, which indicates that CBFs may play a positive role in salt stress tolerance (Li et al., 2011; Yin et al., 2018; Verma et al., 2020).

Little is known about the genetic architecture, molecular mechanism and physiology underlying the salt tolerance of alfalfa compared with other crop species. miR156, which is an important regulator controlling flowering time and plant architecture, has been studied in depth in several plant species (Wang et al., 2009; Wei et al., 2018; Jerome Jeyakumar et al., 2020). Recent studies in alfalfa have shown that miR156 plays a key role in the salt stress response by altering the expression of its target genes, such as *SPL* gene family members (Aung et al., 2015; Arshad et al., 2017a,b, 2018; Feyissa et al., 2021). Other genes such as *MsLEA4-4* (Jia et al., 2020) and *MsCBL4* (An et al., 2020) involved in the salt response have been identified and function in alfalfa.

The use of genome-wide association studies (GWASs) constitutes an effective way to study genetic variations and important complex traits, including those related to salt stress and drought stress tolerance, flowering time and yield in maize, rice and soybean (Qi et al., 2014; Shi et al., 2017; Yu et al., 2017; Zeng et al., 2017; Patishtan et al., 2018; Luo et al., 2019, 2021). In alfalfa, many functional loci associated with salt stress tolerance, drought stress tolerance, forage quality, and disease resistance have been identified *via* GWASs and quantitative trait loci (QTLs) (Yu et al., 2016, 2020; Biazzi et al., 2017; Jia et al., 2017; Liu and Yu, 2017). However, due to the lack of a reference genome, these significantly associated loci can be aligned to the reference genome of only *Medicago truncatula* (Wang et al., 2020). Therefore, some key genes for these traits cannot be found or identified. RNA sequencing (RNA-seq) is a powerful tool for discovering candidate genes and pathways to target traits and has been used widely in many plant (including crop plant) species (Cui et al., 2016; Fang et al., 2017; Yao et al., 2020). The alfalfa reference genomes that have been published in 2020 can greatly improve the accuracy and precision of GWASs and RNA-seq analysis (Chen et al., 2020; Shen et al., 2020; Long et al., 2021).

In this study, we first conducted a GWAS analysis of salt-related traits among a panel that consisted of 220 alfalfa varieties. Then, RNA-seq analysis was used to identify the genes involved in the salt response in alfalfa. Finally, we identified candidate genes associated with salt stress tolerance through a combination of a GWAS and RNA-seq analysis. The results provide valuable information to understand the genetic basis and molecular mechanism of the salt stress response in alfalfa.

## Materials and Methods

### Plant Materials and Phenotypic Analysis

The GWAS panel used in this study consisted of 220 alfalfa varieties from fifty countries throughout the world (Supplementary Table 1). The seeds of this panel were collected from the national grass seed resources of China and the national plant germplasm of the U.S. The plants used for phenotyping in this study were collected from the U.S. National Plant Germplasm System. For tolerance to salt stress, the seedlings of this panel were grown in 20 × 5 cm containers in a greenhouse. The fresh weight of every variety (10 individual plants) under normal conditions and salt-stress conditions was recorded. The tolerance to salt stress of every variety was evaluated by comparing the fresh weight under salt stress with that of an unstressed control; these evaluations were repeated three times for each variety. A relatively high fresh weight suggests that a variety has a relatively high salt tolerance. Germination ability under salt stress was conducted as described in a previous study (Rumbaugh and Pendery, 1990). A relatively high absolute value of germination ability under salt stress suggests that a variety has a relatively high salt tolerance in the germination stage.

For RNA-seq, the Zhongmu No. 1 alfalfa variety was used in this study. Seeds of this variety were placed in a petri dish and subjected to 4°C for 3 days. Then, the seeds were grown in a greenhouse at 24°C (day)/20°C (night) with a 16 h (day)/8 h (night) photoperiod for one week. Afterward, the plants were transplanted into pots and allowed to grow for one week. The alfalfa seedlings were subsequently treated with NaCl solution (300 mM) for 14 days. The roots and leaves of 10 individual alfalfa seedlings under normal conditions and salt-stress conditions were collected. Two replicates were included in this study.

### Genome-Wide Association Study

The genotype of this panel was published in our previous study (Chen et al., 2021). A total of 875,023 high-quality single-nucleotide polymorphism (SNPs) based on the Zhongmu 4 reference haploid genome were used in this study. A GWAS analysis was performed using TASSEL software with the general linear model (GLM) and the mixed linear model (MLM) in conjunction with population genetic structure (Q) and kinship (K), which were analyzed in a previous study (Bradbury et al., 2007; Chen et al., 2021). The thresholds for significantly associated loci were a logarithm of odds (LOD) score ≥ 4.5 (GLM) and 5 (MLM).

### RNA-seq and Transcription Analysis

Total RNA was extracted by using TRIzol reagent (Invitrogen, CA, United States). cDNA library construction and sequencing *via* an Illumina HiSeq 2000 platform were conducted by Novogene (Beijing, China). The clean reads obtained by the standard process were mapped to the Zhongmu No. 1 genome by using TopHat2 software (Trapnell et al., 2012). Only the reads that were unique hits were retained for further analysis. The expected number of fragments per kilobase of transcript sequence per million base pairs sequenced (FPKM) was used to estimate gene expression levels. The Pearson correlation coefficient between replications of different samples was estimated according to the log_10_(FPKM + 1) value. Genes with different expression levels were identified by using DESeq, and Benjamini and Hochberg’s approach was used to control the false discovery rate (FDR) (Benjamini and Hochberg, 1995; Anders and Huber, 2010). Significant DEGs were obtained according to *P*_*adj*_ < 0.05 and | log_2_(fold change [FC])| ≥ 1.

### Candidate Gene Analysis

The reported alfalfa Zhongmu No. 1 and Zhongmu 4 reference haploid genomes were used to identify genes with significantly associated loci. The UniProtKB^1^ and The Arabidopsis Information Resource (TAIR^2^) databases were used for gene annotation. All genes within 200 kb (100 kb up- and downstream) of the significant loci were identified according to the linkage disequilibrium (LD) of the association panel. The genes near significantly associated loci received increased attention. The results of the GWAS and RNA-seq analysis were combined to identify candidate genes for salt stress tolerance in alfalfa.

### Quantitative Real-Time RT-PCR

Quantitative reverse transcription-PCR (RT-PCR) was carried out in triplicate for each sample using SYBR Premix Ex Taq (Takara, Japan) on a 7500 Real-Time PCR System (Applied Biosystems, CA, United States). The gene expression levels in these samples were normalized to that of alfalfa GAPDH, and the 2^–ΔΔCt^ method was used to calculate relative gene expression levels. The primers used in this study are listed in the Supplementary Tables.

## Results

### Analysis of Phenotypic Variation

Phenotypic data of plant tolerance to salt stress and germination ability under salt stress were obtained from the USDA national database^3^. The data showed that the two traits were normally distributed, ranging from 0.599 to 2.616 for tolerance to salt stress and from −0.99 to −0.27 for germination ability under salt stress (Supplementary Figures 1, 2). Correlation analysis showed there was no correlation between the two traits (*r* = −0.01), which means that the genetic mechanisms underlying salt tolerance between the germination and seedling stages are different.

**FIGURE 1:**
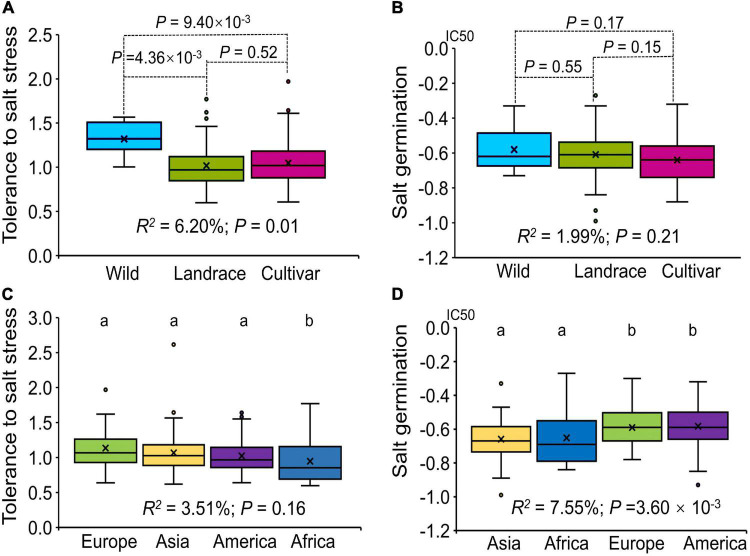
Box plot showing the variation in tolerance to salt stress and germination ability under salt stress evaluated for the association panel. **(A)** Box plot of the tolerance to salt stress among subgroups (Wild, Landrace, Cultivar) according to breeding status. **(B)** Box plot of germination ability under salt stress among subgroups (Wild, Landrace, Cultivar). **(C)** Box plot of the tolerance to salt stress among subgroups (Europe, Asia, America and Africa) according to geographical origin. **(D)** Box plot of germination ability under salt stress among subgroups (Europe, Asia, America and Africa). The different letters above the boxplots in **(C,D)** indicate significant differences at the *P* < 0.05 level according to Duncan’s multiple comparisons test.

Our association panel could be divided into three subgroups according to breeding status: Wild, Landrace and Cultivar. Statistical analysis of the tolerance to salt stress showed that there were significant differences among the three subgroups (*R*^2^ = 6.20%; *P* = 0.01) and that the wild materials had the highest tolerance to salt stress in the seedling stage (Figure 1A). For germination ability under salt stress, there was no significant difference among the three subgroups (*R*^2^ = 1.99%; *P* = 0.21), and compared with the landrace and wild materials, the cultivated materials had a higher germination rate under salt stress (Figure 1B).

The association panel could also be divided into four subgroups according to geographical origin: Asia, Africa, Europe and America. For tolerance to salt stress, there was no significant difference among the four subgroups (*R*^2^ = 3.51%; *P* = 0.16). The alfalfa varieties from the African region had the lowest tolerance to salt stress among the four subgroups (*P* < 0.01, Figure 1C); the European and Asian subgroups showed a higher tolerance to salt stress. For germination under salt stress, there were significant differences among the four subgroups (*R*^2^ = 7.55%; *P* = 3.60 × 10^–3^), and the alfalfa varieties from Asian and African regions had a higher germination rate under salt stress than did the varieties from European and American regions (*P* < 0.01, Figure 1D). Integrating these results, we found that breeding status had a significant effect on tolerance to salt stress at the seedling stage, while geographical origin mainly affected germination under salt stress. In addition, compared with those from the other regions, the alfalfa varieties from the Asian region had a higher tolerance to salt stress at the seedling stage and a higher germination under salt stress, which may be related to the growth environment of alfalfa in Asia.

### Genome-Wide Association Study of Salt Stress Tolerance

Based on the resequencing results, we obtained 875,023 high-quality SNPs distributed across 8 alfalfa chromosomes. The significant SNPs were identified by using the GLM and MLM for tolerance to salt stress and germination ability under salt stress. When the GLM was used, a total of 20 and 24 significant loci were found according to the LOD > 5.00 threshold for tolerance to salt stress (Supplementary Figures 3A,B and Supplementary Table 2) and germination ability under salt stress (Supplementary Figures 3C,D and Supplementary Table 2).

**FIGURE 2:**
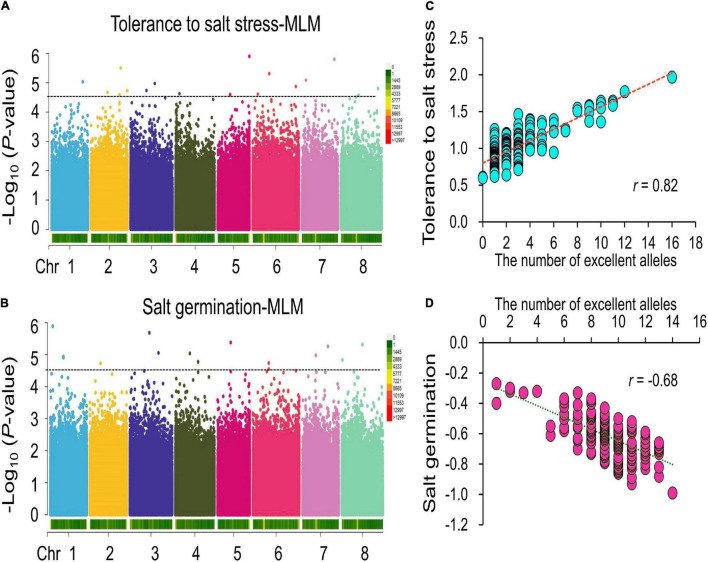
GWAS of tolerance to salt stress and germination ability under salt stress. **(A,B)**, Manhattan plots of the GWAS results of the tolerance to salt stress and germination ability under salt stress among the association panel. **(C,D)**, Correlations between phenotypic traits **(C)** for tolerance to salt stress and **(D)** for germination ability under salt stress and number of excellent alleles identified by the GWAS analysis.

To avoid false-positive results, the MLM was conducted for the two traits. Under the LOD > 4.50 threshold, in total, 18 and 15 significant SNPs were found for tolerance to salt stress and germination ability under salt stress, respectively (Figures 2A,B and Table 1). For tolerance to salt stress, the proportion of phenotypic variance explained (PVE) by the 18 significant SNPs ranged from 12 to 23% (Table 1). The SNP located at 37.34 Mb on chromosome 2 had the largest PVE (*R*^2^ = 23%) for tolerance to salt stress. For germination ability under salt stress, the PVE by the 15 significant SNPs ranged from 9 to 14%, and the SNP located at 29.42 Mb at chromosome 5 had the largest PVE (*R*^2^ = 14%). Taken together, these results provide valuable information for salt tolerance-related gene mining and molecular breeding for alfalfa in the future.

**TABLE 1:** Significant SNPs identified *via* GWAS.

Trait	Marker Name	Chr	Pos	LOD	R^2^	Allele
Tolerance to salt stress	chr5__71576429	5	71576429	5.91	0.18	G/A
	chr7__72989617	7	72989617	5.81	0.19	T/T
	chr2__67122391	2	67122391	5.51	0.18	C/C
	chr6__37606744	6	37606744	5.31	0.15	T/G
	chr7__8960887	7	8960887	5.09	0.14	C/T
	chr1__69958318	1	69958318	5.04	0.14	G/G
	chr3__54403657	3	54403657	4.98	0.14	G/A
	chr6__97595364	6	97595364	4.87	0.13	A/T
	chr8__83555064	8	83555064	4.81	0.16	A/A
	chr3__35880175	3	35880175	4.74	0.13	G/T
	chr2__81709753	2	81709753	4.73	0.13	A/T
	chr2__37337868	2	37337868	4.67	0.23	A/A
	chr4__8427058	4	8427058	4.63	0.13	A/C
	chr6__12007147	6	12007147	4.61	0.13	G/A
	chr5__28059004	5	28059004	4.6	0.13	A/G
	chr2__64256667	2	64256667	4.6	0.13	T/A
	chr8__39543644	8	39543644	4.57	0.13	C/T
	chr8__34586223	8	34586223	4.51	0.12	G/A
Salt germination	chr1__5152907	1	5152907	5.89	0.13	A/A
	chr3__43640366	3	43640366	5.68	0.12	T/T
	chr5__29417598	5	29417598	5.38	0.14	T/T
	chr8__46388623	8	46388623	5.32	0.11	T/T
	chr7__57047224	7	57047224	5.26	0.11	G/G
	chr3__64026087	3	64026087	5.05	0.13	G/A
	chr4__32746445	4	32746445	5.04	0.11	C/C
	chr7__29989074	7	29989074	4.98	0.1	A/A
	chr1__28933639	1	28933639	4.94	0.12	C/C
	chr1__28933708	1	28933708	4.91	0.12	G/G
	chr8__1376482	8	1376482	4.83	0.12	G/G
	chr4__51235577	4	51235577	4.78	0.1	C/C
	chr6__35761890	6	35761890	4.74	0.1	C/C
	chr2__23921553	2	23921553	4.73	0.1	A/A
	chr6__35865758	6	35865758	4.55	0.09	A/A

To further verify the accuracy of the GWAS results, we analyzed the correlations between the number of excellent alleles carried by each material and the two-salt stress-related traits within our association panel. The results showed that the correlation between the number of excellent alleles and tolerance to salt stress was significantly positive, with a coefficient of 0.82 (Figure 2C). For example, Irmsskaja alfalfa from the European region has 16 excellent alleles, and its tolerance to salt stress is 1.97. For germination ability under salt stress, a similar result was found (Figure 2D). The No. 12835 alfalfa from the Asian region has 14 excellent alleles, and its germination ability under salt stress is −0.99, which means that this alfalfa variety has a higher germination under salt stress. These results show that our GWAS results are accurate and can be used for further analysis.

### RNA-seq for Salt Stress Tolerance

**FIGURE 3:**
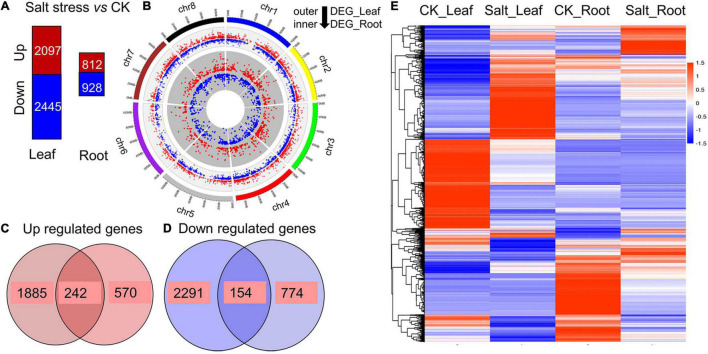
Transcriptome analysis. **(A)** Number of upregulated genes (red) and downregulated genes (blue) between alfalfa seedlings under salt stress and those under normal conditions. **(B)** Distribution of DEGs in the leaves and roots of alfalfa. The inner circle indicates the DEGs in the roots, and the outer circle indicates the DEGs in the leaves. **(C)** Upregulated DEGs common to the leaves and roots. **(D)** Downregulated DEGs common to the leaves and roots. **(E)** Heatmap clustering of all the DEGs according to their expression abundance. The different colors indicate different levels of expression abundance.

We sampled the leaves and roots of 3 Zhongmu No. 1 plants growing for 21 days to construct cDNA libraries and conduct transcriptome sequencing. In total, 2,097 and 812 differentially expressed genes (DEGs) were identified as being upregulated in the leaves and roots, respectively, and 2445 and 928 DEGs were downregulated, respectively (Figure 3A and Supplementary Tables 3–6). These DEGs were distributed across the alfalfa genome, as shown in Figure 3B. Among the 2,909 (2,097 + 812) upregulated DEGs, 242 were common to the leaves and roots under salt stress (Figure 3C and Supplementary Table 7). Among the 3,373 (2,445 + 928) downregulated DEGs, a total of 154 were common to the leaves and roots under salt stress (Figure 3D and Supplementary Table 8). An overview of the expression of these DEGs is shown in a heatmap, and their expression could be divided into six patterns (Figure 3E). Gene Ontology (GO) analysis showed that these DEGs could be divided into three GO categories: biological processes, cellular components and molecular functions (Supplementary Figure 4). In the biological process category, the three largest subcategories were metabolic processes, cellular processes and single-organism processes. For molecular functions, the two most common subcategories were binding and catalytic activity.

**FIGURE 4:**
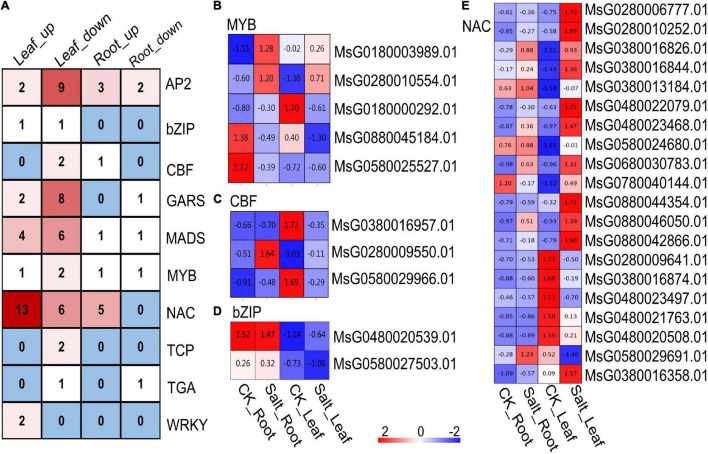
Transcriptome analysis of differentially expressed TFs. **(A)** Number of differentially expressed TFs. **(B)** Differentially expressed MYB-encoding genes. **(C)** Differentially expressed CBF-encoding genes. **(D)** Differentially expressed bZIP-encoding genes. **(E)** Differentially expressed NAC-encoding genes. The color in each cell in panel **(B–D)** indicates the value of expression abundance.

Among these DEGs, many encoding TFs were identified, including the AP2, CBF, NAC and WRKY genes (Figure 4A). A total of eight genes encoding MYB TFs were detected; two of the MYB TF-encoding genes (MsG0180000292.01 and MsG0880045184.01) were downregulated in the leaves under salt stress, and MsG0280010554.01 was upregulated. The expression of the other two genes encoding MYB TFs changed: MsG0180003989.01 was upregulated in the roots under salt stress, and MsG0580025527.01 was downregulated (Figure 4B). Three genes encoding CBF TFs were found, two of which (MsG0380016957.01 and MsG0580029966.01) were downregulated in leaves under salt stress, and another CBF TF-encoding gene (MsG0280009550.01) was upregulated in the roots (Figure 4C). Two genes encoding bZIP TFs were found, and their expression changed only in the leaves. MsG0480020539.01, one of the bZIP TF-encoding genes, was significantly upregulated in alfalfa leaves under salt stress, and MsG0580027503.01, another bZIP TF-encoding gene, was significantly downregulated (Figure 4D). In addition, we found that the expression of 20 NAC genes changed under salt stress. Fourteen NAC genes were upregulated in the leaves, 6 NAC genes were downregulated in the leaves, and 5 NAC genes were upregulated in the roots of alfalfa (Figure 4E). Taken together, these results showed that these TFs may play an important role in alfalfa under salt stress.

### Candidate Gene Analysis for Salt Stress in Alfalfa

**FIGURE 5:**
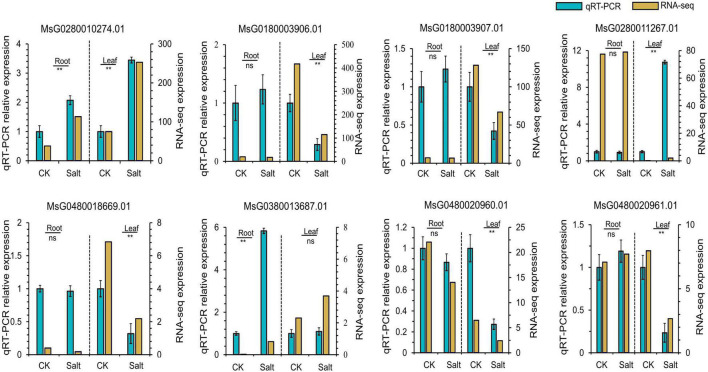
qRT-PCR analysis of eight candidate genes associated with the salt stress response in alfalfa. “**” means a significant difference at the *P* < 0.01 level.

We combined the results of our GWAS and DEG analysis to further analyze the genes associated with salt stress tolerance in alfalfa. According to the LD, a total of 366 candidate genes (211 genes for tolerance to salt stress and 155 genes for germination ability under salt stress) were found for the 33 (18 for tolerance to salt stress and 15 for germination ability under salt stress) significant SNPs (Supplementary Table 9). For the 155 genes associated with germination ability under salt stress, 11 were differentially expressed. Among the 211 candidate genes for tolerance to salt stress, 27 genes were differentially expressed under salt stress. Most of the significant SNPs were located in intergenic spacer regions. To further identify the candidate genes for salt stress in alfalfa, the DEGs closest to the significantly associated SNPs were analyzed by candidate gene association mapping. According to this criterion, there were 5 DEGs associated with tolerance to salt stress and 3 DEGs associated with germination ability under salt stress for analysis (Table 2). To validate the RNA-seq results, we designed primers and performed qRT-PCR on the eight candidate genes (Figure 5 and Supplementary Table 10). The results showed that the RNA-seq results and qRT-PCR results were highly consistent. Among the 8 candidate genes, Msa0033600 (referred to as MsG0180003906.01 in the Zhongmu No. 1 genome annotation), which encodes auxin response protein 28 and is named MsAUX28, was selected for analysis because there were enough SNP sites around this gene.

**TABLE 2:** Candidate genes associated with tolerance to salt stress and germination ability under salt stress.

Traits	SNP	GeneID (Zhongmu-4)	GeneID (Zhongmu No.1)	Annotation
Tolerance to salt stress	chr2__67122391	Msa0216840	MsG0280010274.01	Protein RETICULATA-related
	chr1__69958318	Msa0033600	MsG0180003906.01	AUX/IAA domain
	chr1__69958318	Msa0033660	MsG0180003907.01	AUX/IAA domain
	chr2__81709753	Msa0224870	MsG0280011267.01	ABC transporter-like
	chr4__8427058	Msa0533460	MsG0480018669.01	Leucine-rich repeat
Salt germination	chr3__43640366	Msa0359230	MsG0380013687.01	Leucine-rich repeat
	chr4__51235577	Msa0548430	MsG0480020960.01	Serine-threonine/tyrosine-protein kinase
	chr4__51235577	Msa0545150	MsG0480020961.01	Serine-threonine/tyrosine-protein kinase

**FIGURE 6:**
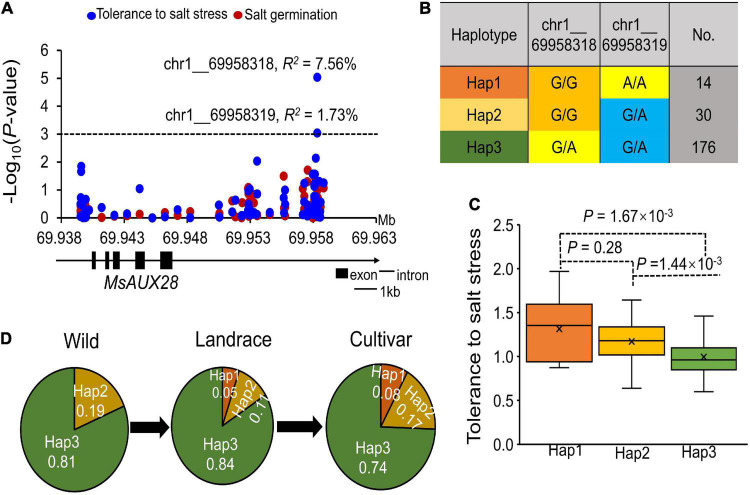
*MsAux28* involvement in the salt stress response in alfalfa. **(A)** Association analysis of the SNPs located within the *MsAUX28* gene region and tolerance to salt stress and germination ability under salt stress. *R*^2^ means the phenotypic variation that can be explained by this locus. **(B)** Haplotype analysis of the two significant SNPs. **(C)** Comparison of salt stress tolerance the three haplotypes. And, there are three haplotypes (Hap1: G/G:A/A; Hap2: G/G:G/A; Hap3: G/A:G/A). **(D)** Allele frequency of the three haplotypes in the Wild, Landrace and Cultivar subgroups.

We found that two SNPs located in the upstream region (∼10 kb) of the MsAUX28 gene were significantly associated with tolerance to salt stress (Figure 6A). The two significant SNPs in the upstream region of *MsAUX28* existed as three different haplotypes in the association panel (Figure 6B). Hap1 (G/G, A/A) had the lowest frequency in the panel but had the highest tolerance to salt stress (Figure 6C). In addition, we found that Hap1 did not exist in the wild alfalfa materials and that the frequency of Hap1 increased in the cultivated materials, which suggests that Hap1 was under selection in the alfalfa breeding process (Figure 6D).

## Discussion

Alfalfa is often planted on saline-alkali land in Asian countries such as China. Therefore, it is necessary to clarify the genetic basis of alfalfa salt tolerance, which could help improve the salt tolerance of this species (Yu et al., 2016). In this study, GWAS analysis based on resequencing was conducted to dissect the genetic architecture of salt stress-related traits, including germination ability under salt stress and tolerance to salt stress. Multiple significant SNPs were identified for these two salt-related traits. Furthermore, several candidate genes involved in salt tolerance were analyzed by a combination of transcriptome analysis and a GWAS. These candidate genes can serve as potential targets of the molecular mechanism of salt tolerance in alfalfa.

Salt stress severely affects the growth and development of alfalfa (Yu et al., 2016; Liu and Yu, 2017). Salt stress causes a decrease in seed germination, plant height and root development in alfalfa (El-Sharkawy et al., 2017; Lei et al., 2018). However, few studies have focused on the effects of salt stress on alfalfa varieties from different geographical regions or on the improvement of breeding status. Based on our results, we found a significant difference in germination ability under salt stress among the alfalfa varieties from different geographical regions. However, there was no difference in this trait among the alfalfa varieties with different breeding status improvements. Compared with those from other geographical regions, the alfalfa varieties from the Asian region had a higher germination ability under salt stress. The reason for this phenomenon may be related to most alfalfa in Asia, especially in China, being planted in saline-alkali soil because most good-quality farmland is used to grow staple crop species such as maize, rice and wheat for human consumption. However, this phenomenon is rare in European and American regions. We also found that, compared with the landrace and cultivated alfalfa, the wild alfalfa varieties were more tolerant to salt stress. These results indicate that many salt tolerance-related genes exist in wild populations. In the future, more salt tolerance-related genes should be mined and transferred to cultivated varieties to improve their salt tolerance. This phenomenon is widespread in other plant species such as maize and rice (Xu et al., 2019; Xu and Sun, 2021).

Many GWASs have been performed on salt stress-related traits in alfalfa. A total of 36 significant markers were found to be associated with salt tolerance during germination under different levels of salt treatments when 198 alfalfa cultivars and landraces were subjected to genotyping by sequencing (GBS) (Yu et al., 2016). Using the same population, Liu and Yu (2017) identified 42 markers significantly associated with salt tolerance-related traits, such as dry weight, plant height, stomatal conductance and leaf chlorophyll content. Due to the lack of a reference genome for alfalfa, these significant markers were aligned only to the reference genome (*Medicago truncatula*) (Wang et al., 2020). Therefore, much important information or presence of significant markers may be lost. In 2020, several reference genomes of alfalfa were published, which allowed the ability to mine candidate genes for salt stress based on GWAS results (Chen et al., 2020; Shen et al., 2020; Long et al., 2021). Moreover, based on the resequencing results, the number of SNPs in this study is more than that in previous studies in which SNPs were identified based on GBS technology (Yu et al., 2016; Liu et al., 2017). In this study, 18 and 15 significant SNPs were identified as being associated with tolerance to salt stress and germination ability under salt stress, respectively. No consistent loci were detected, however, which suggests that there are different genetic structures and molecular mechanisms underlying germination ability under salt stress and salt tolerance in the seedling stage for alfalfa.

Transcriptome analysis has been proven to be a powerful tool for identifying genes associated with plant growth and development. Recently, RNA-seq technology was used to identify key genes related to many important traits in alfalfa, such as fall dormancy (*via* transcriptome profiling of gene expression in fall dormant and non-dormant alfalfa) (Liu et al., 2019), freezing stress (*via* deep-sequencing transcriptome analysis of field-grown crown buds acclimated to freezing stress) (Song et al., 2016) and aluminum stress (*via* a transcriptome analysis of candidate genes potentially involved in the Al stress response) (Liu et al., 2017). In this study, 2,097 and 812 DEGs were upregulated and 2,445 and 928 DEGs were downregulated in the leaves and roots under salt stress, respectively. These genes can help us understand the molecular mechanism of salt tolerance in alfalfa. Previous studies have revealed many genes and pathways controlling salt stress in plants, such as some TF-encoding genes and plant hormone signal pathways (Zhao et al., 2020). We also detected some important TF-encoding genes that were significantly differently expressed under salt stress in alfalfa leaves and roots. In Arabidopsis, the AtMYB49 gene is induced in response to salinity and acts as a positive regulator of salt stress (Zhang P. et al., 2020). MYB TFs are involved in the salt tolerance pathway in many plant species, such as maize, rice and apple (Wang et al., 2014; Tang et al., 2019; Wu et al., 2019). In this study, we also found that MsG0280010554.01, which encodes a MYB TF in the alfalfa genome, was upregulated in the leaves under salt stress. In addition to MYB TFs, NAC TFs also play an important role in plant drought and salt stress responses. For example, *GmSIN1*, which encodes a NAC TF in soybean, promotes root growth and salt tolerance and improves grain yield under salt stress (Li S. et al., 2019). Based on our results, we also found that many NAC TFs were upregulated in alfalfa leaves under salt stress, which suggests that these NAC TFs play an important role in alfalfa salt tolerance.

Many previous studies in other plant species have proven that the combination of GWAS and RNA-seq results can be used as an effective way to identify candidate loci or genes. For example, five important genes were identified for kernel row number in maize by a combination of transcriptome analysis and regional association mapping (An et al., 2019). Seven candidate genes for root-related traits in maize were also identified *via* this strategy (Guo et al., 2019). To our knowledge, this study is the first to use this strategy to identify candidate genes related to salt tolerance in alfalfa. Here, we identified 8 DEGs close to the six significant SNPs (four SNPs located within four DEG sequences and the other two SNPs located in intergenic regions) for germination ability under salt stress and salt tolerance to salt stress.

Plant hormones such as auxin, gibberellic acid (GA), and ABA also play important roles in the salt stress response (Zhao et al., 2020). In rice, *OsIAA20*, which encodes an Aux/IAA protein, improves rice salt tolerance through an ABA-related pathway (Zhang A. et al., 2021). Overexpression of the auxin signaling F-Box 3 receptor (AFB3) can increase salt tolerance in Arabidopsis through the auxin pathway (Garrido-vargas et al., 2020). In tomato, a mutation in *SlARF4*, which encodes an auxin response factor, results in improved tolerance to salt stress and osmotic stress (Bouzroud et al., 2020). Here, we also found that MsG0180003906.01 (*MsAux28*), which encodes an Aux/IAA protein, was significantly associated with salt tolerance in alfalfa. The expression of *MsAux28* was downregulated in alfalfa leaves under salt stress. Therefore, both the overexpression and the knockdown of this gene to validate the function of this gene in alfalfa under salt stress are needed.

In our study, we used a combination of a GWAS and RNA-seq analysis to mine important genes involved in salt tolerance in alfalfa. The results could provide valuable information on the critical regulators of salt tolerance in alfalfa molecular breeding.

## Data Availability Statement

RNA sequence data from roots and leaves after 14 days of salt treatment in a greenhouse has been submitted to The NCBI Sequence Read Archive (BioProject: PRJNA777963). The raw sequencing data can be obtained from the National Genomics Data Center (PRJCA004024, https://bigd.big.ac.cn/).

## Author Contributions

JK and LC designed the study and revised the manuscript. FH, RL, ML, ZW, CW, and YZ analyzed these data and generated the figures and tables. FH and LC analyzed the data and wrote the manuscript. All authors read and approved the final manuscript.

## Conflict of Interest

The authors declare that the research was conducted in the absence of any commercial or financial relationships that could be construed as a potential conflict of interest.

## Publisher’s Note

All claims expressed in this article are solely those of the authors and do not necessarily represent those of their affiliated organizations, or those of the publisher, the editors and the reviewers. Any product that may be evaluated in this article, or claim that may be made by its manufacturer, is not guaranteed or endorsed by the publisher.

## Footnotes

1
https://www.UniProt.org/

2
https://www.arabidopsis.org/

3
https://www.usda.gov/

## Supplementary Material

Supplementary Figure 1 Distribution of germination ability under salt stress among the association panel.

Supplementary Figure 2 Distribution of tolerance to salt stress among the association panel.

Supplementary Figure 3 GWAS analysis of tolerance to salt stress and germination ability under salt stress *via* a GLM.

Supplementary Figure 4 GO analysis of the total DEGs.

Supplementary Table 1 The association panel related information used in this study.

Supplementary Table 2 Significant SNPs identified *via* a GLM.

Supplementary Table 3 Upregulated DEGs in leaves under salt stress.

Supplementary Table 4 Downregulated DEGs in leaves under salt stress.

Supplementary Table 5 Upregulated DEGs in roots under salt stress.

Supplementary Table 6 Downregulated DEGs in roots under salt stress.

Supplementary Table 7 Upregulated genes common to both leaves and roots under salt stress.

Supplementary Table 8 Downregulated genes common to both leaves and roots under salt stress.

Supplementary Table 9 Candidate genes identified *via* GWAS and transcriptome analysis.

Supplementary Table 10 Primers used in this study.
