# Supplementary figures and images for: Genome-Wide Association Analysis Coupled With Transcriptome Analysis Reveals Candidate Genes Related to Salt Stress in Alfalfa (Medicago sativa L.)
Source: Front Plant Sci. 2022 Feb 3;12:826584. doi: 10.3389/fpls.2021.826584 (PMC8850473; doi:10.3389/fpls.2021.826584)

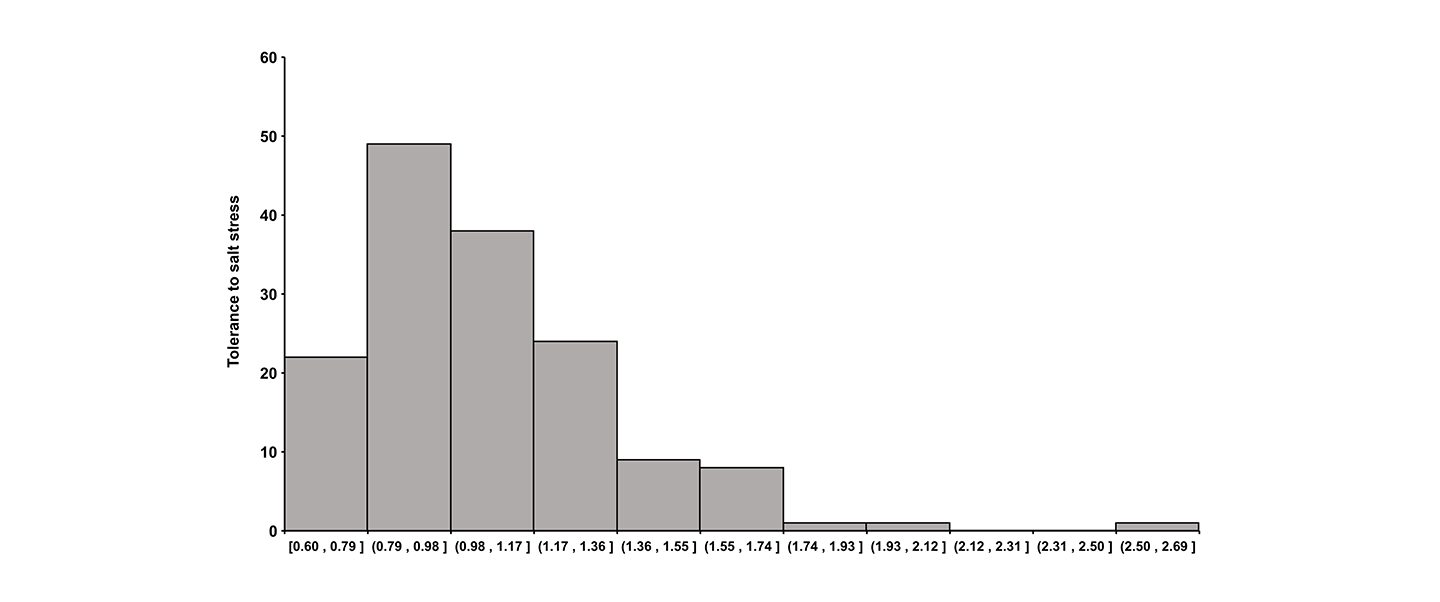

Supplement: Supplementary Figure 1 — Distribution of germination ability under salt stress among the association panel. [file Image_1.TIFF]

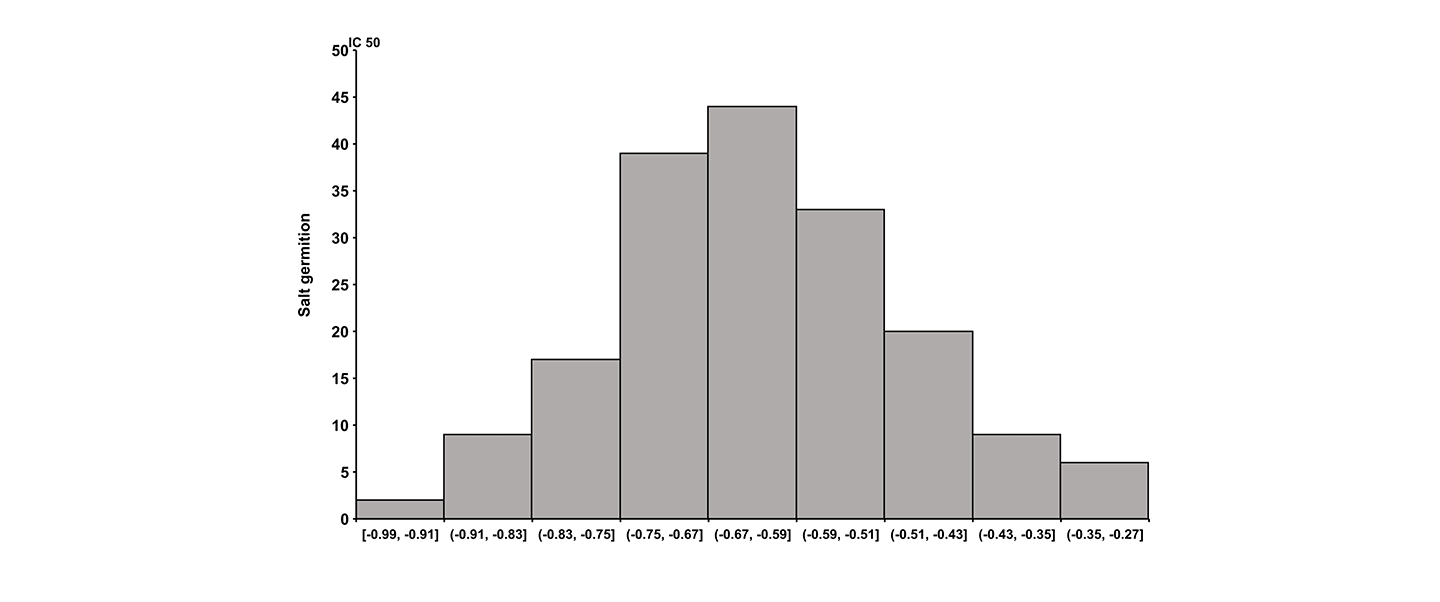

Supplement: Supplementary Figure 2 — Distribution of tolerance to salt stress among the association panel. [file Image_2.TIFF]

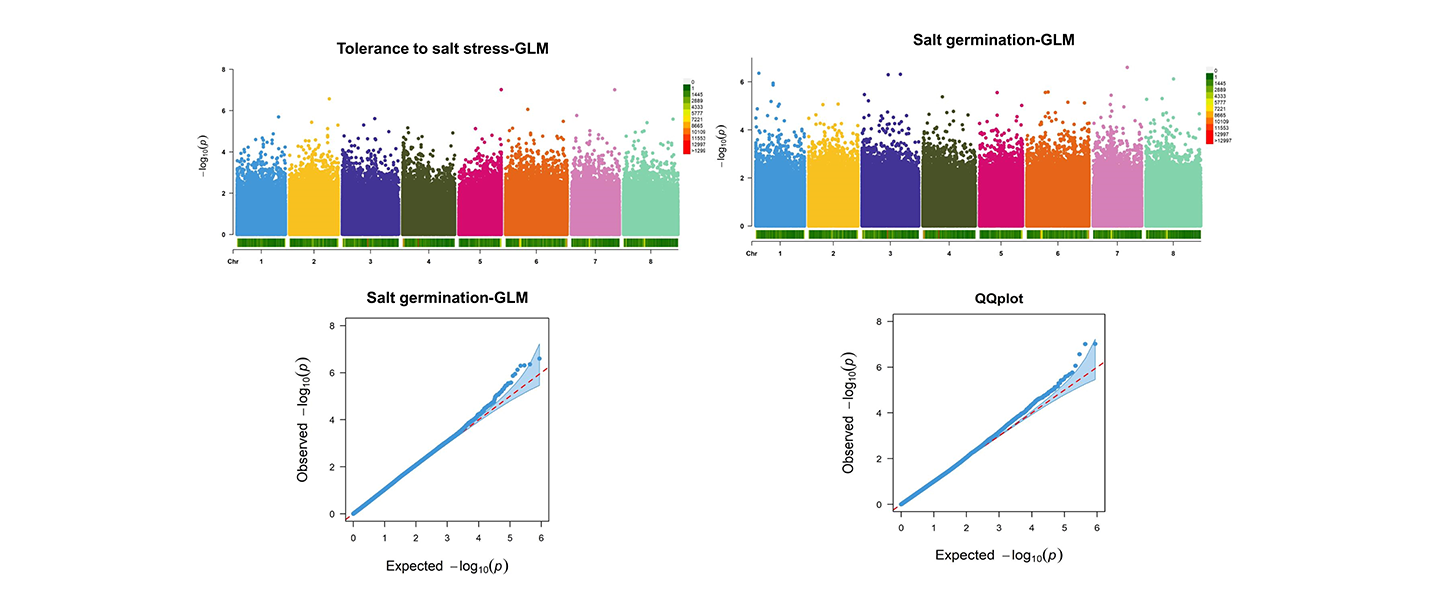

Supplement: Supplementary Figure 3 — GWAS analysis of tolerance to salt stress and germination ability under salt stress via a GLM. [file Image_3.TIFF]

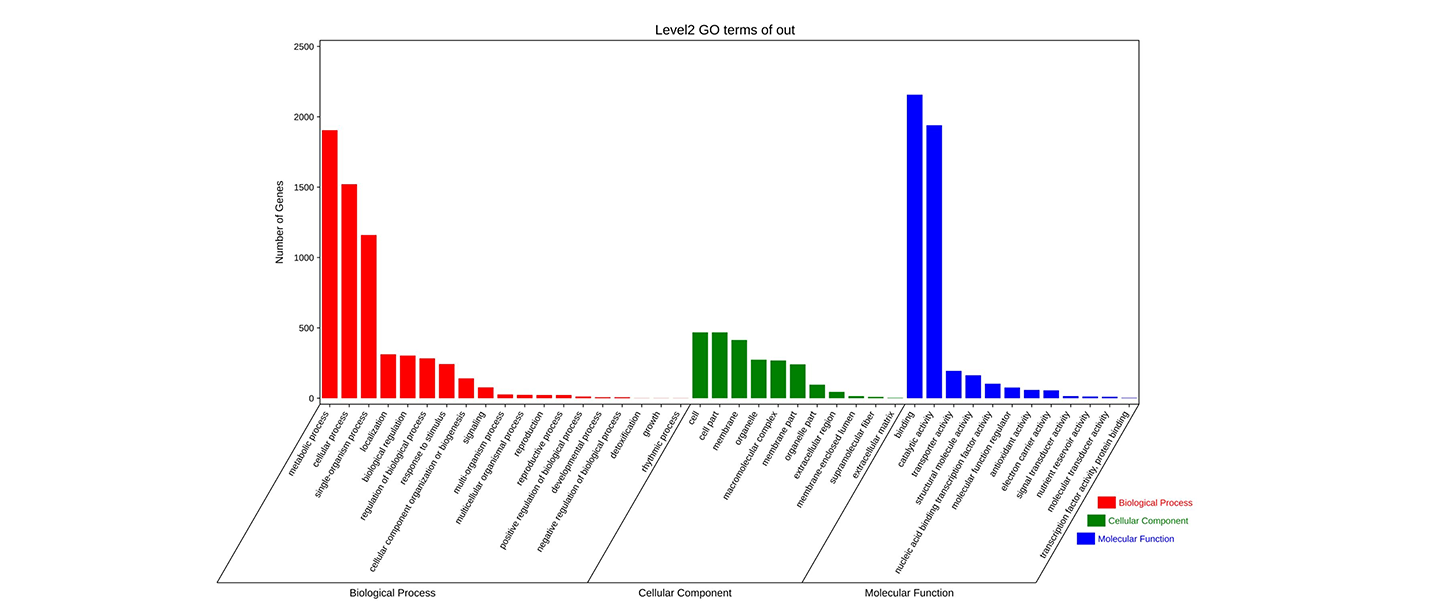

Supplement: Supplementary Figure 4 — GO analysis of the total DEGs. [file Image_4.TIFF]
